# Indian Habits and Diseases

**Published:** 1882-02

**Authors:** 


INDIAN HABITS AND DISEASES.—The following letter, though not written for that purpose, is worthy of record.—[ED.]

UNITED STATES INDIAN SERVICE,
PAPAGO AGENCY, DECEMBER 25, 1881.

MY DEAR PROFESSOR :—As I promised you a letter concerning my new location here in Arizona, I will take to-day, Christmas, as opportunity offers, to write you something of a detailed account of the modes and customs, together with the diseases of my Indian subjects. The Indians among whom I am located are by name the Papagoes, and are members of the large nation known as Pimas, who inhabit this Territory, and also extend far into Mexico. The Papagoes, long years ago, sundered their connection with the great nation on religious accounts; the Papagoes being religiously inclined, and the rest of the nation not. They cut their hair short, and have ever since worn it so. They constitute a large portion of the Indians in this section of the country, and extend several hundred miles into the Mexican territory.

They are preëminently peaceable, and also intelligent; but in war they are great warriors, and are to this day the terror of the Apaches, although they have never been known to be hostile to the white man. They live in rude huts; some of sun-dried brick, others of stone. They are poor farmers, and consequently are insufficiently nourished. The men and women appear well developed, and are manly, fine-looking people. They are not superstitious, and have no traditions that I have been able to learn. Their language is of a most curious character, and no white man, I believe, has ever mastered it. It is almost exclusively guttural.

They are but little inclined to strong drink, which is rather peculiar, I believe. The virtue of their women is actually unimpeachable, and death to both parties is the penalty of adultery or fornication. They are considered the purest Indians in this Western country, while the Pimas, their own countrymen, are given to immorality and drunkenness. There is no syphilis among them, and I have never seen any gonorrhœa yet. Their children are healthy, fat, and fine specimens of youth. There is no scrofula amongst them, although the Mexican children all about here are full of it. I think this is a 'great credit to them.

The diseases of the Indians do not differ greatly from those of the whites. We have here, at certain seasons of the year, the essential fevers; malarial diseases of a varied character; some seasons of contagious diseases; little asthma and catarrh; considerable conjunctivitis, cataract, and other diseases of the eye; some ear troubles. Consumption is rare, and also the other diseases of the East and North. I have not had a chance of observing a single case of cancerous disease since I have been here, nor have I heard them speak of any diseases which might have been it. I believe you mentioned in your lectures that the percentage decreased as we went south. I shall make observations from time to time, and see what the relative amount of cancer is about here.

There are a great many Mexicans about the Reserve, and I think they are the most dissolute, ruthless and good-for-nothing set of people I ever saw. They won't work; don't know how to farm, and are the poorest teamsters, I think, in the world. None could be worse. None of them about here have anything laid up, and are only living on this land by the sufferance of Government.

I have observed a strange disease here, which is quite prevalent among Indian children, and which I forgot to mention. This is a disease known as dirt-eating, and which is mentioned by Dr. Flint in his work on Practice. The children first show a distaste for the mother's milk, refuse it, become crabbed and cross, cry a great deal, and soon they are observed eating dirt. They will continue this practice as long as they are not checked. It is evidently a disease of the digestive system, and probably is due to ill nutrition. The females are very productive, and bear children in quick succession.

There is near here the famous old church of San Xavier, which is supposed to have been built by the Franciscans in 1650. It is a marvel of art for those days, but is now crumbling away quite rapidly. Our dwellings, all through this country, are made of what is known as adobé, or sun-dried brick. They are peculiar-looking edifices. With best regards for yourself, etc., I remain, Yours sincerely,

DR. W. G. COOK.

---

THE HOSPITAL FOR WOMEN, SOHO SQUARE, W. LONDON, DEC. 15, 1881.—A marble bust of Dr. Protheroe Smith, the founder of the Hospital for Women, Soho Square, the first of its specialty, was recently presented to the institution by friends and patients, and was unveiled this day by Sir Rutherford Alcock, K.C.B., the chairman of the committee, on being placed in position in the hall of the hospital. The work has been well executed by Mr. Belt.

